# Evaluation of brain lesion distribution criteria at disease onset in differentiating MS from NMOSD and MOG-IgG-associated encephalomyelitis

**DOI:** 10.1177/1352458518761186

**Published:** 2018-03-07

**Authors:** Jae-Won Hyun, So-Young Huh, Hyun-June Shin, Mark Woodhall, Su-Hyun Kim, Sarosh R Irani, Sang Hyun Lee, Patrick Waters, Ho Jin Kim

**Affiliations:** Department of Neurology, Research Institute and Hospital of National Cancer Center, Goyang, Korea; Department of Neurology, College of Medicine, Kosin University, Busan, Korea; Department of Neurology, Research Institute and Hospital of National Cancer Center, Goyang, Korea; Autoimmune Neurology group, Nuffield Department of Clinical Neurosciences and Oxford University, Oxford, UK; Department of Neurology, Research Institute and Hospital of National Cancer Center, Goyang, Korea; Autoimmune Neurology group, Nuffield Department of Clinical Neurosciences and Oxford University, Oxford, UK; Department of Radiology, Research Institute and Hospital of National Cancer Center, Goyang, Korea; Autoimmune Neurology group, Nuffield Department of Clinical Neurosciences and Oxford University, Oxford, UK; Department of Neurology, Research Institute and Hospital of National Cancer Center, Goyang, Korea

**Keywords:** Multiple sclerosis, neuromyelitis optica spectrum disorder, brain, MRI

## Abstract

**Objectives:**

We aimed to evaluate the utility of the recently described brain lesion distribution criteria to differentiate multiple sclerosis (MS) from aquaporin-4 immunoglobulin G-positive neuromyelitis optica spectrum disorder (NMOSD) and myelin oligodendrocyte glycoprotein immunoglobulin G-associated encephalomyelitis (MOG-EM) at disease onset in an Asian cohort.

**Methods:**

A total of 214 patients who fulfilled the published criteria for MS, NMOSD, or MOG-EM and underwent brain magnetic resonance imaging (MRI) within 3 months of disease onset were enrolled. The brain lesion distribution criteria were defined as the presence of a lesion adjacent to the body of the lateral ventricle and in the inferior temporal lobe, or an S-shaped U-fiber lesion, or a Dawson’s finger-type lesion.

**Results:**

Brain lesions were identified in the initial MRI scans of 166/214 patients. The distribution criteria were applied to these scans (MS (*n* = 94), NMOSD (*n* = 64), and MOG-EM (*n* = 8)). The sensitivity, specificity, and positive and negative predictive values of the criteria for MS versus NMOSD were 79.8%, 87.5%, 90.4%, and 74.7%, and for MS versus MOG-EM these were 79.8%, 100%, 100%, and 29.6%, respectively.

**Conclusion:**

These findings suggest that the brain lesion distribution criteria are helpful in distinguishing MS from NMOSD and MOG-EM in an Asian population, even at disease onset.

## Introduction

Early and accurate discrimination between multiple sclerosis (MS) and neuromyelitis optica spectrum disorder (NMOSD) is an essential practice issue because different therapeutic strategies are provided accordingly.^1^ Recent diagnostic criteria for MS, including the 2010 McDonald and the 2016 Magnetic Resonance Imaging in Multiple Sclerosis (MAGNIMS) criteria, are recommended for patients with suspected MS accompanied by thorough exclusion of other potential causes.^2,3^ However, the assessment of diagnostic biomarkers for exclusion of other disorders mimicking MS, such as aquaporin-4 immunoglobulin G (AQP4-IgG), is not always available in a timely manner and can result in false negatives.^4^ Although typical magnetic resonance imaging (MRI) features that differentiate NMOSD from MS have been well described,^5^ physicians sometimes encounter the patients with borderline radiological presentations, particularly at disease onset. In these cases, supplementary diagnostic indicators can be valuable in preventing inaccurate diagnosis and treatment of MS.

Brain lesion distribution criteria for MS have recently been proposed and are included in the revised diagnostic criteria for NMOSD as “red flags.”^6,7^ The criteria provided localized information of brain MRI lesions suggestive of MS, such as a lesion adjacent to the body of the lateral ventricle and in the inferior temporal lobe, or an S-shaped U-fiber lesion, or a Dawson’s finger-type lesion.^6^ The utility of the criteria in everyday clinical practice across multiple centers has been described;^8^ however, the timing of MRI scans was not focused on early-stage disease and the study involved a predominantly Caucasian population.

We aimed to investigate whether the brain lesion distribution criteria could provide additional clues for differentiating MS from NMOSD and myelin oligodendrocyte glycoprotein immunoglobulin G-associated encephalomyelitis (MOG-EM)―particularly at disease onset―in an independent, large Asian cohort.

## Methods

We enrolled 214 patients at the National Cancer Center (NCC) between 2005 and 2017 who underwent brain MRI scans within 3 months of their first clinical attack (98 fulfilled the McDonald criteria for MS,^2^ 103 patients with NMOSD exhibited positivity for AQP4-IgG and met the 2015 diagnostic criteria for NMOSD,^7^ and 13 patients with MOG-EM). Of the 214 patients, the criteria were selectively applied to the 166 patients who had detectable brain lesions (MS (*n* = 94), NMOSD (*n* = 64), and MOG-EM (*n* = 8)). Patients with a known medical condition that could result in hyperintensity on T2-weighted and fluid-attenuated inversion recovery (FLAIR) sequences were excluded.

AQP4-IgG positivity in NMOSD patients was confirmed using an in-house live cell-based assay at the NCC,^9^ while myelin oligodendrocyte glycoprotein immunoglobulin G1 (MOG-IgG) was tested using a live cell–based assay by the Autoimmune Neurology Group at Oxford University.^10^ The Institutional Review Board of the NCC approved the study protocol and written informed consent was obtained from all participants.

MRI scans were performed using a 1.5- or 3.0-T device, with slice thicknesses varying from 3 to 7 mm. There is no significant difference of slice thickness among the three disease groups (NMOSD, MS, and MOG-EM: 4.7 ± 0.6, 4.7 ± 0.5, and 4.7 ± 0.6 mm, respectively, *p* = 0.905). The brain lesion distribution criteria were defined as at least one lesion with the following characteristic(s): adjacent to the body of the lateral ventricle and in the inferior temporal lobe; juxtacortical S-shaped U-fiber; or Dawson’s finger type (ovoid lesions perpendicular to the lateral ventricle). According to suggestions from a previous lesion probability map study of NMOSD and MS,^11^ border shapes, dimensions, and orientation of Dawson’s finger-type lesion were specified in the definitions of this study. A specified definition of Dawson’s finger-type lesion was used: the lesions had a clear margin and an externally perpendicular orientation from the lateral ventricle, and a diameter ranging from 3 to 19 mm. T2-weighted fast spin-echo and FLAIR sequences, and axial and either coronal or sagittal planes were analyzed for scoring. Evaluation of the lesions according to brain MRI scans was independently performed by a neurologist blinded to the diagnosis (S.-Y.H.) and an unblinded neurologist (J.-W.H.). In case of disagreement, final confirmation was made by a third experienced and blinded neuroradiologist (S.-H.L.).

The presence of brain lesions was compared between patients with MS and NMOSD using Fisher’s exact test. Inter-observer variability was evaluated using Cohen’s kappa, which was ≥0.85 for all criteria. Kappa values for the first, second, third, and the total criteria were 0.91, 0.85, 0.88, and 0.93, respectively.

## Results

### Demographics

The female-to-male ratio (5.1:1 vs 2.3:1 vs 3.3:1) and the mean age of the time of MRI scans at disease onset (36.5 ± 12.1 vs 31.0 ± 9.4 vs 26.7 ± 12.1 years) were higher in NMOSD patients than those in MS or MOG-EM patients, respectively. All patients with NMOSD, MS, and MOG-EM were Asian. At disease onset, 4 of 98 (4%) patients with MS exhibited no initial brain lesion on MRI; however, 39 of 103 (38%) with NMOSD exhibited no initial brain lesion on MRI, a difference that was statistically significant (*p* < 0.001).

### Evaluation of brain lesion distribution criteria

The diagnostic performance of the brain lesion distribution criteria is summarized in Table 1. In patients with initial brain lesions, the sensitivity, specificity, and positive and negative predictive values of MRI brain lesion distribution criteria were 79.8%, 87.5%, 90.4%, and 74.7%, when differentiating MS from NMOSD, and 79.8%, 100%, 100%, and 29.6%, when discriminating MS from MOG-EM, respectively. The criteria demonstrated 79.8% sensitivity, 88.9% specificity, a positive predictive value (PPV) of 90.4%, and a negative predictive value (NPV) of 77.1% for the differentiation of MS from all patients with NMOSD as well as MOG-EM.

**Table 1.** Diagnostic performances of brain lesion distribution criteria for differentiating MS from NMOSD and MOG-EM.

	Number of patients	Criterion (a): lesions adjacent to the body of a lateral ventricle and in the inferior temporal lobe	Criterion (b): U-fiber lesions	Criterion (c): Dawson’s finger type lesions	Full criteria (a, b, or c)
NMOSD, *n*	64	3	7	0	8
MS, *n*	94	53	25	62	75
Sensitivity		56.4	26.6	66.0	79.8
Specificity		95.3	89.1	100	87.5
Positive predictive value		94.6	78.1	100	90.4
Negative predictive value		59.8	45.2	66.7	74.7
MOG-EM, *n*	8	0	0	0	0
MS, *n*	94	53	25	62	75
Sensitivity		56.4	26.6	66.0	79.8
Specificity		100	100	100	100
Positive predictive value		100	100	100	100
Negative predictive value		16.3	10.4	20.0	29.6

MS: multiple sclerosis; NMOSD: neuromyelitis optica spectrum disorder; MOG-EM: myelin oligodendrocyte glycoprotein immunoglobulin G-associated encephalomyelitis.

When differentiating MS from NMOSD, the sensitivity and specificity were the highest for the Dawson’s finger-type lesion criterion, but the lowest for the juxtacortical S-shaped U-fiber lesion criterion. The same results were observed when distinguishing MS from MOG-EM.

### Exceptional cases in MS and NMOSD

**Figure 1.**
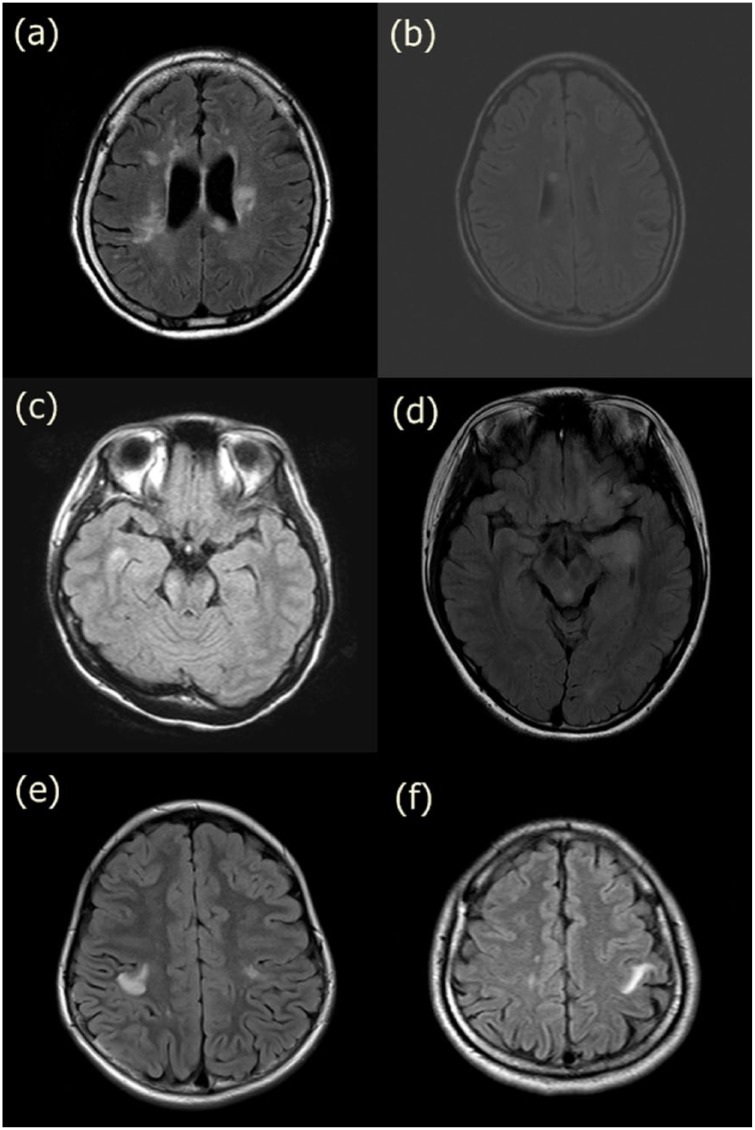
MRI findings of exceptional cases in neuromyelitis optica spectrum disorder: (a) and (b) lesions adjacent to the body of the lateral ventricles, (c) and (d) lesions in the inferior temporal lobes, and (e) and (f) juxtacortical lesions involving U-fiber.

Nineteen of 94 (20.2%) patients with MS did not fulfill the brain lesion distribution criteria. These patients exhibited brainstem and/or subcortical white matter lesions. Eight of 64 (12.5%) patients with NMOSD met the criteria, in whom the juxtacortical S-shaped U-fiber lesion was most commonly observed (*n* = 7), followed by the criterion including lateral ventricle and inferior temporal lesions (*n* = 3). Some patients with NMOSD exhibited Dawson’s finger-like lesions (Figure 1(a) and (b)), but none with NMOSD fulfilled the specified definition of Dawson’s finger-type lesions. MRI findings in NMOSD patients satisfying the criteria are presented in Figure 1. None of the patients with MOG-EM fulfilled the criteria at the onset of disease.

## Discussion

This study demonstrated that the brain lesion distribution criteria can be useful in differentiating MS from NMOSD and MOG-EM, even at disease onset, in a large cohort of Asian patients with idiopathic central nervous system inflammatory diseases.

The previously proposed brain lesion distribution criteria were based on lesion probability maps of brain MRI scans of patients with MS and NMOSD, performed at a mean disease duration of 12.3 ± 6.8 years in MS patients and 8.44 ± 6.4 years in NMOSD patients.^6^ In a recent validation,^8^ however, the difference in mean age at onset to first MRI scan was 4 years between MS and NMOSD patients. When we differentiated patients with MS and NMOSD, the sensitivity (79.8% vs 90.9%) was slightly lower, but specificity (87.5% vs 87.1%) was similar to previously reported results. This may have been influenced by the early timing of MRI scans in this study because lesion load may be lower at disease onset than at more than 4 years’ disease duration. The inter-observer agreement was excellent in this study (kappa ≥ 0.85) and even higher than a previous validation (kappa = 0.76).^8^ Considering the importance of early diagnosis and treatment in neuroinflammatory diseases, this study is meaningful to confirm an additional value of brain lesion distribution criteria at disease onset, which enabled the distinction of MS from NMOSD and MOG-EM.

The previous validation of brain lesion distribution criteria was based on a western Caucasian population,^8^ whereas this study was based on an Asian population. In a previous study, the ratio of MS to NMOSD was approximately 2:1 in a western Caucasian population;^6^ in our cohort, this ratio was approximately 1:1. In our case, PPV would decrease and NPV would increase; thus, the lower PPV in this study than in the previous studies (PPV: 90.4% vs 90.9%–97.9%) was a reasonable outcome. The NPV in this study (NPV: 74.7% vs 86.2%–87.1%) was also lower than in previous studies, which may be explained by the early timing of MRI scans, when the likelihood of false negatives would be higher.

The definition of Dawson’s^12^ finger-type lesion primarily relied on the pathology findings of MS; however, concerns regarding its vagueness have risen. Using a specified definition, including detailed morphological information, consistent with a previous study,^6^ no patients with NMOSD or MOG-EM exhibited Dawson’s finger-type lesion, and a reliable inter-observer agreement was noted in this study.

Previous studies have reported that the absence of brain lesions on MRI is a favorable finding for the diagnosis of NMOSD rather than MS.^5,13^ We reconfirmed that normal brain MRI features provide clues to distinguish NMOSD from MS, even at the early stage of disease.

This study had several limitations. The majority of initial brain MRI scans were performed before the patients were referred to our center; consequently, the MRI protocols could not be fully unified. Nevertheless, we could apply the criteria across hospitals and MRI scanners, which reflected the real-world clinical practice. In addition, patients with AQP4-IgG- and MOG-IgG-negative MS/NMOSD-like syndrome were not included because we could not confirm the pathological diagnosis in this group; hence, we could not evaluate the diagnostic performance of the criteria in these patients.

In conclusion, when patients with marginal clinical manifestations at disease onset are encountered, the brain lesion distribution criteria can provide additional clues to differentiate MS from NMOSD and MOG-EM. If the patient does not fulfill these criteria, careful clinical assessments combined with validated laboratory investigations, such as AQP4-IgG and MOG-IgG, should be considered.
